# Supplementary material for: Obesity and accelerated epigenetic aging in a high-risk cohort of children
Source: Sci Rep. 2022 May 18;12:8328. doi: 10.1038/s41598-022-11562-5 (PMC9117197; doi:10.1038/s41598-022-11562-5)
Supplement: Supplementary file 1 — Supplementary Information. [file 41598_2022_11562_MOESM1_ESM.docx]

Supplemental Materials

| Supplementary Table 1. Summary Statistics for Currently Assembled Cohort | | | |  |
| --- | --- | --- | --- | --- |
| **Variable, mean (SD)** | **Total (N=439)** | **Maltreatment (N=373)** | **Comparison (N=66)** | **p-val** |
| Body Mass Index (BMI) | 21.6(5.8) | 22.0(5.9) | 19.9(4.8) | .0009** |
| Age (at collection) | 11.4(1.4) | 11.5(1.4) | 11.1(1.4) | .08 |
| Biological Sex M(F) | 51.5%(48.5) | 51.5%(48.5) | 51.5%(48.5) | .99 |
| Income $10,000/year | 3.7(3.3) | 3.3(3.1) | 5.8(3.8) | <.0001*** |
| Race |  |  |  |  |
| Black | 15.0% | 15.6% | 12.1% | .47 |
| White | 71.3% | 70.2% | 77.3% | .24 |
| Other | 13.7% | 14.2% | 11.7% | .43 |
| Ethnicity |  |  |  |  |
| Hispanic | 13.2% | 14.8% | 4.6% | .02* |
| *†P<.10, *P<.05, **P<.01, ***P<.0001* | |  |  |  |

| **Supplemental Table 7.** z-scored BMI predicted by maltreatment-status | | |  |  |  |
| --- | --- | --- | --- | --- | --- |
| Outcome: BMI | Model 1 | Model 2 | Model 3 | Model 4 | Model 5 |
|  | Est.±SE | Est.±SE | Est.±SE | Est.±SE | Est.±SE |
| Parameter |  |  |  |  |  |
| Maltreatment | 0.37(.14)** | 2.87(1.29)* | 2.53(1.38)† | 0.30(.12)* | 0.21(.13) |
| Age | -- | 0.27(.07)*** | 0.35(.06)*** | 0.17(.03)*** | 0.18(.03)*** |
| Sex (Male) | -- | -- | 0.59(.23)** | -0.26(.09)** | -.26(.09)** |
| Race/Ethnicity (Ref=white/non-Hispanic) |  |  |  |  |  |
| Black | -- | -- | -- | 0.10(.12) | 0.01(.12) |
| Other | -- | -- | -- | -0.09(.15) | -0.12(.15) |
| Hispanic | -- | -- | -- | 0.09(.16) | 0.06(.16) |
| Household Income | -- | -- | -- | -- | -0.04(.01)** |
| *^†^P<.10, *P<.05, **P<.01, ***P<.0001* |  |  |  |  |  |
